# Metachromatic Leukodystrophy: New Therapy Advancements and Emerging Research Directions

**DOI:** 10.1212/WNL.0000000000213817

**Published:** 2025-06-27

**Authors:** Marije A.B.C. Asbreuk, Daphne H. Schoenmakers, Laura Ann Adang, Shanice Beerepoot, Caroline Bergner, Annette Bley, Jaap Jan Boelens, Marianna Bugiani, Valeria Calbi, Àngeles García-Cazorla, Erik A. Eklund, Francesca Fumagalli, Sabine Weller Grønborg, Samuel Groeschel, Peter M. Van Hasselt, Carla E.M. Hollak, Simon A. Jones, Tom J. de Koning, André B.P. van Kuilenburg, Lucia Laugwitz, Caroline Lindemans, Fanny Mochel, Andreas Øberg, Dipak Ram, Ludger Schöls, Caroline Sevin, Jigyasha Sinha, Frédéric M. Vaz, Ayelet Zerem, Nicole I. Wolf

**Affiliations:** 1 Department of Child Neurology, Emma's Children's Hospital, Amsterdam UMC location Vrije Universiteit, the Netherlands;; 2 Amsterdam Leukodystrophy Center, Amsterdam Neuroscience, Cellular & Molecular Mechanisms, the Netherlands;; 3 Medicine for Society, Platform at Amsterdam UMC location University of Amsterdam, the Netherlands;; 4 Children's Hospital of Philadelphia, PA;; 5 Department of Neurology, University of Pennsylvania, Philadelphia;; 6 Princess Máxima Center for Pediatric Oncology, Utrecht, the Netherlands;; 7 Leukodystrophy Center, Department of Neurology, University Hospital Leipzig, Germany;; 8 University Children's Hospital, University Medical Center Hamburg Eppendorf, Hamburg, Germany;; 9 Department of Pediatrics, Stem Cell Transplant and Cellular Therapies, Memorial Sloan Kettering Cancer Center, New York, NY;; 10 Department of Paediatrics/Child Neurology, VU University Medical Centre, Amsterdam Neuroscience, the Netherlands;; 11 Department of Pathology, VU University Medical Centre, Amsterdam Neuroscience, the Netherlands;; 12 San Raffaele Telethon Institute for Gene Therapy (SR-TIGET), Milan, Italy;; 13 Pediatric Immuno-Hematology Unit, IRCCS Ospedale San Raffaele Milan, Italy;; 14 Neurometabolic Unit, Neurology Department, Hospital Sant Joan de D'eu, Barcelona, Spain;; 15 Pediatrics, Clinical Sciences, Lund University, Sweden;; 16 Unit of Neurology, IRCCS Ospedale San Raffaele Milan, Italy;; 17 Center for Inherited Metabolic Diseases, Department of Pediatrics and Adolescent Medicine and Department of Clinical Genetics, Copenhagen University Hospital Rigshospitalet, Denmark;; 18 Neuropediatrics, General Pediatrics, Diabetology, Endocrinology and Social Pediatrics, University of Tübingen, University Hospital Tübingen, Germany;; 19 Department of Metabolic Diseases, University Medical Center Utrecht, the Netherlands;; 20 Department of Endocrinology and Metabolism, Amsterdam UMC location University of Amsterdam, the Netherlands;; 21 Genomic Medicine, Manchester University NHS FT, United Kingdom;; 22 Department of Pediatrics, Lund University, Sweden;; 23 Department of Neurology and Genetics, University of Groningen and University of Medical Center Groningen, the Netherlands;; 24 Laboratory Genetic Metabolic Diseases, Department of Clinical Chemistry, Amsterdam UMC, Vrije Universiteit Amsterdam, Amsterdam Gastroenterology & Metabolism, the Netherlands;; 25 Neuropediatrics, General Pediatrics, Diabetology, Endocrinology and Social Pediatrics, University of Tuebingen, University Hospital Tübingen, Germany;; 26 Institute for Medical Genetics and Applied Genomics, University of Tübingen, Germany;; 27 Department of Pediatric Hematopoietic Stem Cell Transplantation, UMC Utrecht and Princess Maxima Center, the Netherlands;; 28 Regenerative Medicine Institute, University Medical Center Utrecht, the Netherlands;; 29 Reference Center for Adult Leukodystrophy, Department of Medical Genetics, Sorbonne University, Paris Brain Institute, La Pitié-Salpêtrière University Hospital, France;; 30 Norwegian National Unit for Newborn Screening, Division of Pediatric and Adolescent Medicine, Oslo University Hospital, Norway;; 31 Department of Pediatric Neurology, Royal Manchester Children's Hospital, United Kingdom;; 32 Department of Neurology and Hertie-Institute for Clinical Brain Research, University of Tübingen, Germany;; 33 German Center of Neurodegenerative Diseases (DZNE), Tübingen, Germany;; 34 Reference Center for Leukodystrophies, Pediatric Neurology Department, Hôpital Bicêtre, Le Kremlin Bicêtre, France;; 35 Department of Pediatric Neurology, Center of Neurosciences, Narayana Hospital, RN Tagore Hospital, Kolkata, India;; 36 Laboratory Genetic Metabolic Diseases, Department of Laboratory Medicine and Pediatrics, Emma Children's Hospital, Amsterdam UMC location University of Amsterdam, the Netherlands;; 37 Amsterdam Gastroenterology Endocrinology Metabolism, Inborn Errors of Metabolism, the Netherlands;; 38 Core Facility Metabolomics, Amsterdam UMC location University of Amsterdam, the Netherlands; and; 39 Pediatric Neurology Institute, Leukodystrophy Center, Dana-Dwek Children's Hospital, Tel Aviv Sourasky Medical Center, Faculty of Medicine, Tel Aviv University, Israel.

## Abstract

Metachromatic leukodystrophy (MLD) is a rare autosomal recessive lysosomal storage disorder caused by disease-causing variants in the gene coding for arylsulfatase A, leading to deficient enzyme activity and subsequent accumulation of sulfatides. MLD is characterized by demyelination and neurodegeneration of the central and peripheral nervous system, manifesting as progressive motor and cognitive defects in affected individuals. This review provides a comprehensive overview of the significant progress made in MLD research in the past decade, regarding natural history, disease and treatment mechanisms, and newborn screening (NBS). Traditionally, MLD has been classified according to age at onset (late-infantile, early-juvenile and late-juvenile, and adult MLD), with earlier forms leading to more rapid neurologic decline. New data show that the type of presenting symptoms further influences the dynamic of disease progression. Patients with a cognitive presentation have a much slower or even no motor decline than patients with a mixed motor and cognitive presentation. Research advancements have enabled improved understanding of the effects of allogeneic hematopoietic stem cell transplantation and the development of novel therapeutic approaches, including hematopoietic stem cell gene therapy, which is now authorized in the EU, United Kingdom, and United States as treatment for selected patients with early-onset forms of MLD. Both hematopoietic stem cell transplantation and hematopoietic stem cell gene therapy are most effective when administered before disease onset. To identify presymptomatic patients, NBS for MLD is becoming available in several countries, resulting in new challenges. Decisions regarding patient eligibility for these treatments in already symptomatic individuals, as well as the timing of treatment for patients identified through NBS, require thorough understanding of disease progression. Biomarkers may be helpful for disease staging and prediction of disease evolution. Moreover, apart from timing, challenges remain regarding optimal treatment strategies across MLD subtypes, especially late-onset MLD, and management of the clinical heterogeneity and course of the disease. Another important issue is ensuring therapy accessibility, which forms a substantial barrier for equitable care. Continued research and international collaboration are essential to address these challenges, with the goal of improving care and outcomes for patients with MLD and their families.

## Introduction

Metachromatic leukodystrophy (MLD, OMIM #250100) is a rare lysosomal storage disorder caused by biallelic disease-causing variants in *ARSA*, the gene encoding arylsulfatase A (ASA), leading to decreased or absent activity of ASA. Deficiency of this enzyme results in the inability to degrade sulfate-containing glycosphingolipids called sulfatides, which accumulate in the CNS and peripheral nervous system (PNS), primarily in membrane structures, and causes neurodegeneration. MLD is usually classified into 4 subtypes, depending on age at onset: late-infantile (onset before 30 months), early-juvenile (onset between 2.5 and 6 years), late-juvenile (onset between 7 and 16 years), and adult (onset at 16 years or older), although categories are somewhat arbitrary and definitions vary.^1^ Birth prevalence of MLD has been reported to range between 0.16 and 1.85 per 100,000 live births.^2^ Certain populations have a higher birth prevalence, as high as 1 per 75 live births in Habbanite Jews, 1 of 2,500 live births in the Yup'ik ancestry from Alaska, and 1 in 6,400 live births in the western Navajo Nation.^3^

Late-infantile MLD is rapidly progressive, leading to loss of all gross motor functions before 40 months of age and death usually within 5 years after onset of symptoms, but survival may be longer with symptomatic care.^1,3^ Juvenile and adult MLD progress more slowly, but motor deterioration accelerates once independent walking is lost.^3^ Early stages often involve behavioral problems and psychiatric symptoms.^3^ Especially, the adult onset is, therefore, often mistaken for early-onset dementia or a psychiatric disorder due to predominant cognitive deficits and psychiatric symptoms. Survival for both juvenile and adult MLD is variable. Patients with juvenile onset often do not reach 20 years; life expectancy in the adult form ranges from several years to decades.^3^

**Figure 1:**
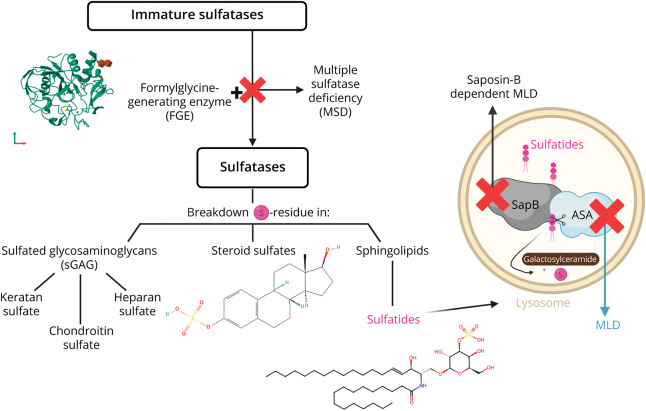
Degradation of sGAGs, Steroid Sulfates, and Sulfatides In metachromatic leukodystrophy (MLD), variants in the ARSA gene or deficiency of the saposin B cofactor (encoded by PSAP) leads to the accumulation of sulfated sphingolipids called sulfatides in the nervous system. In multiple sulfatase deficiency (MSD), variants in the SUMF1 gene impair formylglycine-generating enzyme (FGE), inactivating all sulfatases and causing buildup of sulfated glycosaminoglycans (sGAGs), steroid sulfates, and sulfatides. Created in BioRender. Wolf N (2025). BioRender.com/yw8bb6d.

Biallelic disease-causing variants in the *PSAP* gene, which encodes the protein activator saposin B (SapB), cause SapB-dependent MLD (OMIM #249900).^4^ Biallelic disease-causing variants in the *SUMF1* gene, a gene essential for posttranslational modification of sulfatase enzymes, results in multiple sulfatase deficiency (MSD, OMIM #272200) (Figure 1).^4,5^ In MSD, sulfatides as well as other sulfated molecules, including steroid sulfates and glycosaminoglycans, accumulate, creating a biochemical profile that, for sulfatides, resembles MLD. Sulfatide accumulation in MLD, MSD and SapB-dependent MLD, destabilizes the myelin sheath, leading to demyelination. Distinguishing SapB-dependent MLD and MSD is crucial for treatment decisions because gene therapy options are still not available for these diseases. Hematopoietic stem cell transplantation (HSCT) is not an option in MSD, and it is unclear whether HSCT may be beneficial in the early stages of SapB-dependent MLD.^6^

MLD is often suspected when typical abnormalities on brain MRI are found and confirmed by measuring ASA activity in leukocytes (or fibroblasts) and sulfatide levels in urine and genetic testing.^4^ It is also important to evaluate for ASA pseudodeficiency, which is present in 1%–2% of Caucasians and up to 17.4%–25.6% of the Tunisian population.^7,8^ In ASA pseudodeficiency, ASA activity when measured in vitro is significantly reduced (5%–15% of normal enzyme levels) due to 2 *ARSA* variants, without clinical manifestations. The most frequent pseudodeficiency allele results in the loss of 1 of 3 N-glycosylation sites, causing the loss of a downstream polyadenylation signal, which leads to instable mRNA and thus lower protein expression. Although independently insufficient to result in disease, it is not fully understood how the pseudodeficiency alleles affect function of ASA when in cis with a disease-causing variant.^9^

Over the past decade, significant progress has been made in the understanding of MLD, especially regarding treatment options and disease mechanisms. This narrative review provides an overview of recent developments and addresses knowledge gaps, highlighting key challenges and outlining future research directions.

## Clinical Presentation

### Cognitive vs Motor Presentation of MLD

While classification of MLD subtypes according to age at first symptoms is universally accepted, the type of presenting symptoms is linked to disease progression.^1^ Three distinct symptom clusters have been recognized: motor, cognitive, and combined motor-cognitive.^1^ A presentation with predominantly motor symptoms and signs, seen particularly in late-infantile patients, includes gross motor delay, abnormal gait, and/or tremor and pyramidal signs. The cognitive presentation, common in late-onset MLD, encompasses symptoms such as irritability, attention deficit, or learning difficulties, as well as psychiatric changes including behavioral regression, mood disorders, and personality changes. Typically, onset is insidious and progression slow, with normal neurologic examination, which can delay diagnosis of a leukodystrophy for years.^10^

A study categorized 97 patients with all MLD subtypes by early symptoms, motor, cognitive, or combined motor-cognitive.^1^ For early-onset MLD, first symptoms were reported to be motor or combined motor-cognitive, with rapid disease progression. In the juvenile and adult forms, the type of presenting symptoms (motor vs cognitive) rather than the age at onset was predictive of disease progression. Patients with motor symptoms at onset had a significantly faster disease progression than those with cognitive symptoms alone. Once motor deterioration started, rapid regression followed, regardless of age at onset. This was confirmed by another study, which showed that when patients with late-infantile MLD could walk independently before disease onset, bulbar dysfunction and loss of trunk control appeared 6 months later than when this milestone was not reached.^11^ Moreover, delay in reaching developmental milestones is possible before first clear MLD-related symptoms, especially in late-infantile patients.^10^

## Genotype-Phenotype Association

There are some *ARSA* variants with a well-established association with the MLD subtype. Biallelic protein-truncating variants (PTVs) lead to minimal or no residual ASA activity and consequently result in early-onset MLD, for example, the most common late-infantile variant (c.465+1G>A).^12^ The c.1283C>T, p.(Pro428Leu) variant is associated with juvenile or adult-onset MLD, with a motor presentation, and occurs frequently in Central and Western Europe.^13^ Non-Caucasian patients often harbor novel *ARSA* variants that are categorized as variants of unknown significance (VUSs).^13^ A recent study combined in vitro ASA activity from expression of VUSs in cell cultures with clinical information extracted from 489 published cases.^14^ This defined a classification system of *ARSA* variants according to their association with clinical subtype. For example, variants associated with late-infantile MLD and all PTVs are categorized as severe, and any variant that occurs in combination with a severe variant in a patient with adult MLD is categorized as mild. This approach resulted in a 76% accurate severity prediction but still has its limitations. Other factors can also influence the phenotype, illustrated by phenotypic variability in juvenile siblings.^15^ This means that correct prognostication remains challenging in patients with hitherto undescribed variants.

## Pathophysiology

### Sulfatides

Sulfatides are glycosphingolipids characterized by variations in the lengths of their N-linked fatty acids (C16–C26), hydroxylation, and sphingoid base. Sulfatides are predominantly found in myelin, expressed in oligodendrocytes in the CNS and Schwann cells in the PNS.^5^ Outside the nervous system, sulfatides are abundant in the kidney, gastrointestinal tract, islets of Langerhans, and trachea.^5^ They play a critical role in various biological functions: insulin secretion, immune response, hemostasis/thrombosis, cancer, and infections.^5^

During human development, the composition of the N-linked fatty acid in the CNS sulfatides shifts from predominantly containing C16-C18 fatty acids in early infancy to predominance of C24 fatty acid–containing sulfatides with myelination.^16^ Animal studies support this, showing that immature oligodendrocytes contain sulfatides with long-chain fatty acids while mature oligodendrocytes contain very long–chain fatty acids.^17^ In adult myelin sheaths, sulfatides primarily contain very long–chain fatty acids (C22–C26), whereas astrocytes and neurons have predominantly sulfatides with shorter chains (C16–C18), reflecting biological diversity of sulfatide species.^5^ These variations in fatty acid length are believed to contribute to differences in membrane rigidity and, therefore, membrane dynamics across cell types.^17^

Sulfatides are essential in the nervous system, initiating myelin formation in the PNS and maintaining myelin integrity in both CNS and PNS.^5^ In the CNS, they play additional regulatory roles by inhibiting oligodendrocyte differentiation and restricting myelin-associated axon outgrowth.^5^ Sulfatides also facilitate the transport of proteolipid protein 1, the major myelin protein, and support myelin membrane adhesion through myelin and lymphocyte protein clustering glycosphingolipids during myelin maturation.^5^ Furthermore, sulfatides are vital for glial-axon signaling; in their absence, sodium and potassium ion channel clusters are abnormally localized and maintained at the nodes of Ranvier, affecting proper signaling conduction.^5,16^

**Figure 2:**
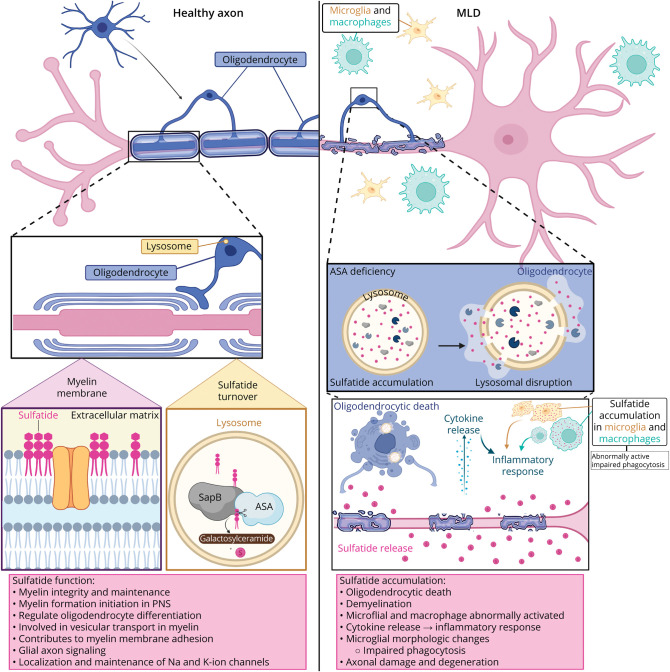
Functions of Sulfatides in the CNS Sulfatides, which are critical for myelin integrity and maintenance, are embedded in the myelin membrane. In healthy oligodendrocytes, sulfatides undergo a turnover process in the lysosome, where saposin B (SapB) facilitates the interaction between the enzyme arylsulfatase A (ASA) and the sulfatide, which hydrolyses sulfatides into sulfate and galactosylceramide, maintaining a balance within the cell and myelin sheaths. In metachromatic leukodystrophy (MLD), ASA deficiency leads to accumulation of sulfatides in lysosomes of different cell types. This accumulation eventually causes the lysosome to rupture, releasing its contents into the oligodendrocyte, leading to cell death and demyelination. Sulfatide release induces secretion of cytokines and triggers a nitric oxide–mediated cell stress response, which initiates an inflammatory reaction. Created in BioRender. Wolf N (2025). BioRender.com/sifofjr.

Excess sulfatides trigger in vitro pathologic inflammatory responses in glial cells, leading to morphological changes in microglia and induction of inflammation-associated molecules, including nitric oxide and cytokines.^18^ These inflammatory effects are believed to be partly mediated through an L-selectin–dependent mechanism, which becomes dramatically downregulated on exposure to sulfatides. Sulfatides released from demyelination into the extracellular space may function as endogenous inflammatory agents and autoantigens, engaging the immune system of the brain.^18^ Figure 2 presents an overview of sulfatide roles in the healthy CNS compared with MLD.

Altered sulfatide levels are also observed in other neurologic diseases.^19^ Elevated sulfatide concentrations in the gray matter (GM) were reported in Parkinson disease (PD) while significant sulfatide loss occurs in both the cerebral GM and white matter (WM) in Alzheimer disease.^19,20^ In multiple sclerosis, reduced sulfatide levels and impaired remyelination contribute to progressive myelin loss with changes in lipid distribution correlating with local morphological changes in mouse models.^19^ These examples demonstrate the need for tightly regulated sulfatide levels because both excess and deficiency may disrupt critical biological processes, underscoring the essential roles of sulfatides in myelin integrity.

### α-Synuclein

The protein α-synuclein is mainly found in neurons, and its aggregation is believed to play an essential role in the pathogenesis of Lewy body diseases, including PD. Identified risk genes associated with Lewy body disease include *GBA1*, the causative gene for Gaucher disease; *GALC*, when deficient leading to Krabbe disease; and *SMPD1*, for which defects result in acid sphingomyelinase deficiency. Recently, *ARSA* has been postulated as an additional genetic modifier for PD. Knockout *ARSA* cell lines show α-synuclein aggregation, probably due to loss of the cytosolic molecular chaperone function of ASA.^21^ A recent preprinted study compared *postmortem* brain tissue from 5 patients with MLD, 2 with juvenile and 3 with late-infantile onset, with that of 5 matched controls. Juvenile-onset patients exhibited intraneuronal α-synuclein aggregation in the dentate nucleus and globus pallidus. By contrast, α-synuclein presence in late-infantile patients was inconsistent, with significant aggregation only in the dentate nucleus.^22^ These preliminary findings suggest that α-synuclein pathology in MLD may be an additional factor in GM pathology.

## Therapy: New Developments

### Hematopoietic Stem Cell Transplantation

Allogenic HSCT is considered the standard treatment for presymptomatic and early-symptomatic adult and late-juvenile MLD. Its effects for patients with early-onset MLD are limited.^23^ After treatment, initial progression of MRI abnormalities and symptoms may occur, probably due to chemotherapy neurotoxicity and the time until full brain engraftment is achieved, but long-term clinical stabilization and even improvement of WM changes are possible when treatment is administered in presymptomatic or early stages.^23^ Despite improvements in the past decades, transplant complications such as graft vs host disease (GvHD), infections, toxicity, chronic graft rejection, and malignancy risk are not rare.^24^ The mortality rate in patients with MLD is still approximately 10%–15% but has been reported to be higher in some cohorts.^23,24^

**Figure 3:**
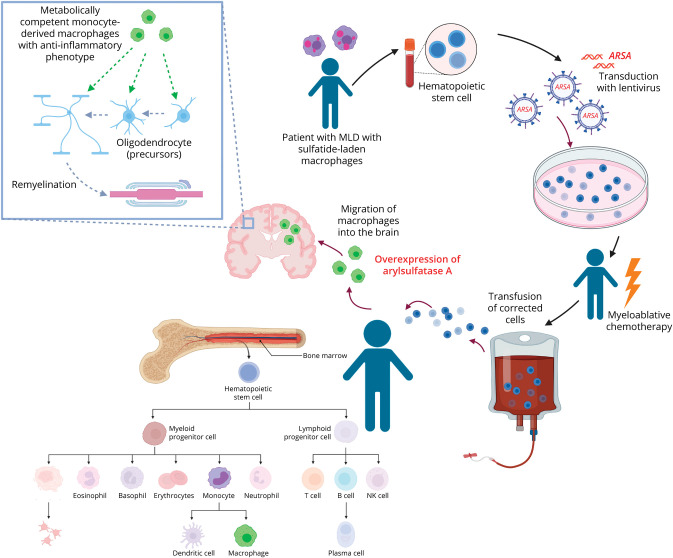
Ex Vivo Gene Therapy for MLD Patient hematopoietic stem cells are collected and treated ex vivo with a lentiviral vector coding for *ARSA* cDNA, leading to supranormal expression of ASA. After myeloablative chemotherapy, patients are infused with treated hematopoietic stem cells. Monocyte-derived metabolically competent macrophages migrate to the brain where they are able to digest sulfatides. Their anti-inflammatory effects are supposed to enable oligodendrocyte differentiation and remyelination. ASA = arylsulfatase A; cDNA = complementary DNA; MLD = metachromatic leukodystrophy. Created in BioRender. Wolf N (2025). BioRender.com/n59r016.

The treatment effect of HSCT was long hypothesized to be mediated by cross-correction (Figure 3), assuming that normal ASA would be transferred from donor-derived macrophages/microglia to ASA-deficient glial and neuronal cells although this has only been demonstrated in a mouse model treated with HSC-gene therapy (HSC-GT).^25^ There are several arguments against cross-correction as the sole factor for HSCT success. First, ASA endocytosis and routing to the lysosomes is mediated by a mannose-6-phosphate receptor-dependent mechanism.^26^ However, ASA secreted by macrophages and microglia was shown to lack mannose-6-phosphate, hampering its uptake by recipient cells.^27^ In a postmortem study involving 2 patients with MLD treated with HSCT who died from disease progression, ASA was identified in donor macrophages in the CNS, in a granular lysosomal pattern, confirming presence of ASA in the CNS after transplantation. ASA could not be detected in oligodendrocytes and astrocytes, either because it was below the detection threshold or because there was no relevant cross-correction. Metabolically competent donor macrophages were not only able to digest sulfatides but further expressed anti-inflammatory markers, which may support oligodendrocyte survival and differentiation compared with nontransplanted patients. Supporting this hypothesis, higher numbers of oligodendrocytes and decreased g-ratio in transplanted patients indicated remyelination.^28^ Of interest, a study in a mouse model for Krabbe disease showed similar results. HSCT restored the beneficial function of GALC-expressing CNS macrophages independent of cross-correction, leading to a reduction in galactosylceramide accumulation and disease burden by decreased neuroinflammation due to donor cells with normal GALC activity.^29^

Despite an initial successful treatment with HSCT, long-term neurologic and motor deterioration leading to death has been observed.^23,24^ This is paralleled by peripheral neuropathy and progressive brain atrophy, but without MLD-typical WM involvement on MRI, even when treated early.^30^ In 1 successfully transplanted patient who died from graft vs host disease, donor macrophages expressing ASA were present in the WM, but not in GM structures. Possible reasons are that GM harbors fewer macrophages/microglia than WM, or that myelin debris, predominately found in damaged WM, acts as a strong attractor of donor macrophages/microglia.^31^

### Hematopoietic Stem Cell and Progenitor Ex Vivo Gene Therapy

Atidarsagene autotemcel (arsa-cel) is an autologous HSC post-lentiviral gene therapy ex vivo (HSC-GT) to treat presymptomatic late-infantile and early-juvenile and early-symptomatic early-juvenile MLD patients.^32^ CD34^+^ cells are harvested, engineered ex vivo with a lentiviral vector encoding human *ARSA* cDNA, and infused after busulfan conditioning, leading to overexpression of ASA and higher-than-normal ASA activity.

Safety and efficacy were evaluated by comparing early-onset HSC-GT–treated MLD patients with a historical cohort of 31 untreated patients.^32^ Results were confirmed by a long-term study on efficacy and safety, which also showed that HSC-GT is influenced by MLD subtype, patient age, and timing of treatment.^33^ Compared with HSCT, HSC-GT was better tolerated, without GvHD, graft failure, or need for long-term immunosuppression.

Clinical trials with follow-up durations up to 7.5 years confirmed that HSC-GT prevents severe motor and cognitive impairments and slows disease progression.^32,34^ When presymptomatic at the time of intervention, most treated patients had normal cognitive development compared with untreated patients. Most patients acquired motor skills, either within the range of healthy children or had stabilized motor performance. Furthermore, central and peripheral demyelination and brain atrophy were either prevented or delayed. HSC-GT has, therefore, been approved by regulatory agencies in Europe and the United Status and marks a significant advancement in therapeutic options for patients with early-onset MLD. To date, patients have maintained improved ASA activity with stable disease. Its application in late-juvenile MLD is currently being further investigated (NCT04283227).

### Intracerebral Gene Therapy

One of the disadvantages of HSCT and HSC-GT is the delayed delivery of ASA-expressing cells. Therefore, more efficient delivery methods are needed, also able to target glial cells and neurons. Intracerebral injections of a viral vector (AAVrh.10-hARSA) have been tested in nonhuman primates and were safe and effective. In a phase I-II clinical trial, 3 patients with late-infantile MLD (2 presymptomatic and 1 early-symptomatic) and 1 early-juvenile early-symptomatic patient, aged 9 months to 5 years, were treated with this vector.^35^ AAVrh10-hARSA was detected in urine, and significant increase in ASA activity was observed in the CSF. However, all patients had clinical and radiologic disease progression similar to or more rapid than the natural history of MLD. T2 hyperintense areas developed around the injection sites on brain MRI.^35^ Why this approach failed in humans is not known. Of interest, similar MRI changes were seen in participants in an intracerebral gene therapy trial for Sanfilippo disease, shown to be due to extracellular spilling of lysosomal enzymes with subsequent WM damage.^36^

### Enzyme Replacement Therapy

IV enzyme replacement therapy (ERT) stabilizes or improves non-CNS symptoms of lysosomal storage diseases. MLD mouse model studies demonstrated improvements in motor and behavioral symptoms, prompting further investigation in a clinical trial.^37^ To assess the safety and efficacy of IV recombinant human (rh)ASA, dose-escalated IV rhASA was administered every 14 days for 52 weeks to 13 patients with MLD with an onset ≤4 years.^38^ No serious adverse events related to the treatment were reported. While peripheral nerve pathology did not worsen, motor and cognitive functioning continued to deteriorate, suggesting that IV rhASA does not cross the blood-brain barrier in therapeutic quantities. Nonetheless, the results concerning peripheral nerves indicate that rhASA may have some positive effects on patients with MLD, perhaps due to better penetration through the (homeostatic) blood-nerve barrier, which is leakier than the blood-brain barrier.^38,39^

Intrathecal administration of rhASA was, therefore, evaluated as an alternative to IV ERT in a clinical trial involving 24 children with MLD who had an onset ≤30 months conducted over 38 weeks.^40^ Different dosages ranging from 10 mg to 100 mg were tested across several cohorts. No serious adverse events related to rhASA were reported, although 25% of patients experienced adverse events associated with the intrathecal device or drug delivery method. An overall decline in motor function was observed over time, but patients receiving the highest dose showed the least pronounced decline.^40^ The treatment was well tolerated, leading to an extension to evaluate long-term safety (NCT01887938). In addition, an ongoing phase 2b trial investigated the effects of a higher dose of 150 mg weekly of rhASA in 36 patients with MLD (NCT03771898). This dose corrected biochemical defects, and delayed pathologic features seemed to delay neurologic deterioration and structural changes in some children.^41^ However, a lack of efficacy on the primary end point (gross motor function) led to discontinuation of this treatment strategy.

## Symptomatic Care

In most patients with MLD, the disease is too advanced at the time of diagnosis to benefit from treatment.^42^ For these patients, treatment focuses on symptomatic care to maximize comfort, requiring a multidisciplinary approach.^43^ Treatment for spasticity can include physical therapy or medications such as intrathecal baclofen or botulinum toxin.^44^ The gallbladder is significantly affected by sulfatide accumulation, with an elevated risk of gallbladder carcinoma.^45^ Therefore, screening for gallbladder abnormalities and early preventive laparoscopic cholecystectomy are recommended, including in patients treated with HSC-GT or HSCT.^45^ Some patients experience abdominal pain or discomfort, whereby gallbladder removal may also be considered. Behavioral issues and cognitive decline, particularly in patients with late-juvenile and adult MLD, pose a particular challenge.^46^

The disease burden of MLD is high and increases as the disease progresses.^47^ Specific clinical symptoms found to be troublesome by caregivers across all types are immobility and respiratory difficulties.^47^ A systematic literature review revealed that the emotional burden is high for caregivers and 71% experiences anxiety and depression.^47^ In addition, it ascertained that parents of children with early-onset MLD reported significantly lower health-related quality of life compared with parents of healthy children.^47^

## Biomarkers for MLD

### Brain MRI

Brain MRI scans in patients with MLD show diffuse T2 hyperintense WM signals with T2 hypointense dots or stripes, depending on image orientation, leading to tigroid appearance typical of this disease.^48^ In the early stages, U-fibers and cerebellar WM are spared. Motor tracts are spared in patients with cognitive presentation.^11^ Brain MRI in late-infantile patients, even with clinical CNS involvement, may be (almost) normal, causing diagnostic delay.^48^ In the later onset forms, MRI abnormalities always precede clinical symptom onset.^48^ The MLD-MRI severity score is used to semiquantify brain MRI involvement by analyzing the extent of WM involvement and atrophy, using a 0–34-point system adapted from adrenoleukodystrophy, and correlates with IQ and motor function.^49^

The volume of abnormal WM T2 hyperintensities has been established as an additional MRI biomarker for MLD, termed “demyelination load,” and shown to correlate with motor and cognitive function.^50^ Of interest, there is evidence of cases whereby pathologic T2 hyperintense WM signal in late-infantile MLD may return to normal after the age of 4 years, with abnormal WM microstructure, probably due to storage material and tissue gliosis.^51^

**Figure 4:**
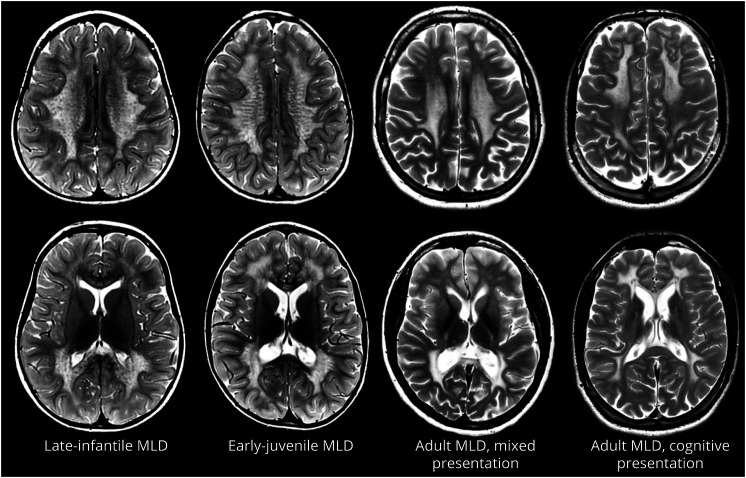
MRI Changes in Relation to Age at Onset and Type of Presentation Axial T2-weighted MRI scans of 4 patients with MLD, at diagnosis. (A) Late-infantile MLD: age 2 years, (B) early-juvenile MLD: age 5 years, (C) adult MLD (mixed presentation): age 24 years, and (D) adult MLD (cognitive presentation): age 20 years. Note the diffuse white matter involvement in early-onset MLD, with the typical tigroid pattern and the sparing of the subcortical fibers. In the late-infantile patient, the parieto-occipital white matter is more affected than the frontal white matter, which also illustrated more severely abnormal splenium vs the genu of the corpus callosum. In the cases with adult onset, there is, already at diagnosis, more pronounced atrophy than in the patient with early-juvenile MLD. The tigroid pattern is less evident. Note the diffuse white matter involvement with posterior predominance in the patient with a mixed cognitive and motor presentation and the sparing of the motor tracts and a frontal predominance in the patient with cognitive presentation and otherwise normal motor function and neurologic examination. MLD = metachromatic leukodystrophy.

Significant atrophy occurs in later stages but may be present at diagnosis, especially in late-juvenile and adult cases (Figure 4). GM atrophy, particularly cerebral cortical GM volume loss, is present in the early stages of the disease and linked to reduced motor function.^49,50^ GM volume loss was found in both treated and untreated patients with MLD with a positive correlation with motor and cognitive decline.^30^

### Quantitative Brain MRI Measures

A study in 2018 used quantitative magnetic resonance spectroscopy to compare 21 patients with MLD (12 juvenile, 9 adults) with 16 control participants, evaluating the metabolic differences between these groups.^52^ N-acetyl-aspartate (NAA), a marker of neuroaxonal integrity, was identified as the most significant prognostic parameter because of its strong association of baseline NAA concentrations with motor function at follow-up. Another study confirmed this finding, showing positive correlations for NAA levels in the deep WM with motor and cognitive functioning.^53^ Patients with poor treatment outcomes had severely decreased concentrations of NAA and glutamate, along with increased levels of lactate and myo-inositol already at baseline.^52^ Moreover, MLD spectra are characterized by elevated levels of choline-containing compounds, associated with active demyelination.^52^ Diffusion parameters are another parameter with prognostic value, especially in highly organized brain structures as the corticospinal tracts. Apparent diffusion coefficient (ADC) and fractional anisotropy correlated with gross motor function and IQ, and clearly abnormal ADC values in the central region predict rapid disease progression at diagnosis in patients with no or mild motor involvement.^54^ In the future, new MRI methods sensitive to early WM changes may help to detect the onset of brain disease activity already in presymptomatic individuals.

### Neurofilament Light

Neurofilament is a scaffolding protein primarily found in myelinated axons, supporting conduction speed and structural integrity. Although present in dendrites and the neuronal soma, it is most abundant in myelinated axons. Elevated neurofilament levels in CSF and blood indicate axonal injury. Neurofilament light (NfL), most prevalent and soluble neurofilament subunit in both CSF and blood, is a key biomarker for different neurologic diseases.^55^ Significantly elevated blood NfL levels were observed at diagnosis in presymptomatic and symptomatic patients with MLD and correlated with MRI abnormalities.^55^ Rapid disease progression in late-infantile and early-juvenile patients was associated with higher NfL levels. Despite decreased levels during follow-up in both transplanted and untreated patients, NfL levels remained elevated compared with controls, reflecting both acute and chronic axonal damage in patients with MLD.^55^

### ASA Activity

Development of an improved test to measure ASA activity was needed because the measurement of leukocytes and fibroblast lysates suffered from multiple pitfalls.^7^ Therefore, liquid chromatography–tandem mass spectrometry (LC-MS/MS) assays have been developed to measure ASA activity in leukocytes and dried blood spots (DBSs).^7^ A refined fluorometric ASA assay resulted in better discrimination of MLD subtypes than traditional assays, with a residual ASA activity below 1% found in early-onset MLD.^12^ It was hypothesized that ASA activity varies between patients with a cognitive, motor, or mixed presentation. Recent results showed that patients with pure cognitive and behavioral onset exhibited higher ASA enzyme activity.^1^ ASA activity in a high-throughput cellular model system was also used to test pathogenicity of *ARSA* variants and association with MLD subtypes, with good success as to predicting pathogenicity, while MLD subtypes could not be reliably predicted for all tested variants.^14^

## Newborn Screening

Newborn screening (NBS) for patients with MLD has the potential to facilitate early identification before development of symptoms, enabling timely treatment and thereby improved outcomes. This is particularly valuable as MLD is typically diagnosed after clinical onset and only occasionally through family testing. However, NBS also presents uncertainties, especially regarding the ethical implications of early diagnosis when the onset may be late, or the *ARSA* variants are novel. In the latter case, disease variant prediction with enzyme analysis may assist phenotype prediction, but not with full certainty. European consensus-based recommendation and a disease-specific guideline for the United States were recently published, addressing these questions.^4,43^ Both studies support implementation of NBS programs for MLD, recommending timely communication and support to NBS-diagnosed patients and families. Early-onset MLD is advised to be treated with HSC-GT and late-onset MLD, for the time being, with HSCT because HSC-GT is not yet approved for the latter. The therapeutic window to treat patients with an anticipated onset in adulthood remains a challenge and is ideally discussed case-by-case in an expert panel. Pretreatment and post-treatment monitoring is advised for future guideline optimization.

Over the past 5 years, a combination of 2 biochemical assays using DBS has been developed for NBS: (1) sulfatides, (2) ASA enzyme activity, followed by genetic testing.^4^ The feasibility of MLD screening using these assays was evaluated in a recent retrospective study involving more than 27,000 newborns.^56^ To minimize false-positive rates, a 3-tier screening approach was implemented. In the first tier, C16:0-sulfatides in DBS were quantified using ultraperformance LC-MS/MS. Newborns with abnormal C16:0-sulfatide levels (n = 195) were further screened with the second tier by measuring ASA activity in DBS. The third tier is a genetic test to confirm the MLD diagnosis. Of the 27,000 newborns, 2 high-risk cases were identified who turned out to be heterozygous carriers of a single disease-causing *ARSA* variant. The specificity was nearly 100% with second-tier testing.^56^

A 2024 study explored the potential of using an additional short-chain sulfatide, 16:1-OH, in the first-tier screening.^57^ The quantification of 16:1-OH sulfatide resulted in a 20-fold reduction in first-tier positive results requiring second-tier screening, significantly lowering the false-positive rate without inducing false-negative results. Therefore, simultaneous measurement of both 16:0 and 16:1-OH species is recommended for optimization of the first-tier sulfatide assay. Application of this strategy in a pilot 3-tier NBS study involving 109,259 newborns in Germany led to the identification of 2 early-onset cases and 1 late-onset case with no false-positive results.^58^ Both early-onset patients received HSC-GT, and the patient with late-onset MLD is scheduled to undergo HSCT at a later age.

## Disparities Regarding Diagnosis and Treatment Accessibility

Underdiagnosis of rare diseases is more common among minority ethnic and racial groups, including leukodystrophies.^59^ Sex-based discrimination also affects the diagnosis of leukodystrophy, with male patients being 1.35 times more likely to receive a diagnosis compared with female patients.^59^ Limited access to diagnostic tools contributes to the issue of underdiagnosis. Basic diagnostic methods such as MRI or biochemical/genetic testing are difficult to access in many countries creating additional barriers for early treatment.

Significant work is still needed to improve treatment accessibility, even in Europe. A web-based European Union (EU) survey showed that HSCT is only available in 23 of 30 countries in Europe for MLD, with 1–3 treatment centers per country.^6^ Consensus guidelines for treatment initiations are not universally followed, with symptomatic late-infantile patients receiving HSCT in some European centers. The availability of HSC-GT is limited to qualified treatment centers, which all accept international referrals. In the EU, regulation on cross-border patient care allows patients to travel to other EU countries for treatments with limited availability, fostering access to innovative treatments. Still, those treatments and indications are not universally known. To improve access to expert advice independent from country of residence, the MLD initiative and the European Reference Network “Rare Neurological Diseases” have, therefore, established a European treatment eligibility board to discuss treatment indications and advice for newly diagnosed patients with MLD, with the possibility of ad hoc meetings for urgent cases.^60^

Even when available, the high costs of HSCT and HSC-GT present a substantial barrier. Currently, HSC-GT is reimbursed in the United States and many European countries, primarily in Scandinavia and Western Europe. This uneven distribution of treatment accessibility leads to inequitable care, as patients' access to life-saving therapies depends on geographic location and socioeconomic status. Global health initiatives and policies aimed at improving health care equity are essential for addressing these disparities.

## Discussion

Over the past decades, researchers and clinicians have made significant progress in understanding and treating MLD. Ongoing research continues to enhance our understanding of the pathologic mechanisms behind disease progression and mode of action of these therapies.

Treatment of patients with late-juvenile and adult MLD remains challenging. While HSC-GT represents a major advancement for late-infantile and early-juvenile MLD, a clinical trial is still ongoing to confirm its efficacy and safety for late-onset juvenile patients (NCT04283227). The efficacy of HSC-GT for patients with adult MLD is not yet being investigated. Although ongoing research has advanced our understanding of the mechanisms supporting the use of HSCT, significant challenges remain such as the toxicity of chemotherapy and GvHD. These issues limit its appeal as a treatment option. Nevertheless, HSCT remains a cornerstone therapy for presymptomatic or early-symptomatic late-onset MLD.

NBS reliably identifies patients with MLD, enabling presymptomatic treatment, which leads to more beneficial treatment outcomes. It also allows genetic counseling of identified families at risk. However, it poses challenges as well, such as emotional and psychological distress in families with predicted late onset of MLD or when phenotype prediction is ambiguous. Determining the optimal timing for treatment in late-onset forms remains unresolved—is it better to wait for evidence of disease onset, for example, on MRI (and potentially improved therapies) or initiate treatment long before the expected symptomatic age? When NBS detects a disease-causing *ARSA* variant with an uncertain disease progression, counseling of families and subsequent patient care become more complex. Ongoing efforts to develop and refine prognostic biomarkers or algorithms are important to improve the ability to predict outcomes and distinguish clinically relevant variants at birth.

Complexities surrounding the availability of resources for diagnosis and treatment of MLD highlight critical disparities in health care systems worldwide. Significant barriers in many countries complicate timely diagnosis. The available treatment options often lack reimbursement, preventing their use. Initiatives as the MLD Treatment Eligibility Panel allow rapid access to multicenter expert opinion. International collaboration and investment in health care infrastructure, particularly in underserved regions, are essential for improving access to both diagnostic tools and treatments of MLD.

## Conclusion

Recent research in MLD has led to novel treatments and improved outcomes. The emergence of HSC-GT and advances in HSCT are promising for altering the disease trajectory, especially when applied early. NBS will be a critical step toward timely diagnosis to achieve optimal treatment results. Moreover, new biomarkers hold promise to improve the assessment of disease progression in patients with MLD. However, despite these advancements, significant challenges remain, including the need for individual treatment recommendations because of clinical disease heterogeneity. Disparities in treatment accessibility continue to pose a substantial barrier to equitable care. Assessment of the long-term effectiveness of new therapies requires further research. Future research should also focus on deepening our understanding of the pathophysiology of MLD and on identifying new biomarkers to improve delineation of the optimal therapeutic window. Ongoing innovation remains necessary to improve the quality of life and outcomes for patients with MLD and their families.

## Glossary

ADC: apparent diffusion coefficient
ASA: arylsulfatase A
DBS: dried blood spot
ERT: enzyme replacement therapy
GM: gray matter
GvHD: graft vs host disease
HSCT: hematopoietic stem cell transplantation
HSC-GT: HSC-gene therapy
LC-MS/MS: liquid chromatography–tandem mass spectrometry
MLD: metachromatic leukodystrophy
MSD: multiple sulfatase deficiency
NAA: N-acetyl-aspartate
NBS: newborn screening
NfL: neurofilament light
PD: Parkinson disease
PNS: peripheral nervous system
PTV: protein-truncating variant
SapB: saposin B
VUSs: variants of unknown significance
WM: white matter
